# An Amino Acid Deletion in *SZT2* in a Family with Non-Syndromic Intellectual Disability

**DOI:** 10.1371/journal.pone.0082810

**Published:** 2013-12-06

**Authors:** Michelle Falcone, Kemal O. Yariz, David B. Ross, Joseph Foster, Ibis Menendez, Mustafa Tekin

**Affiliations:** 1 Dr. John T. Macdonald Department of Human Genetics and John P. Hussman Institute for Human Genomics, Miller School of Medicine, University of Miami, Miami, Florida, United States of America; 2 Comprehensive Neurobehavioral Institute, Plantation, Florida, United States of America; Innsbruck Medical University, Austria

## Abstract

Autosomal recessive intellectual disability (ID) is characterized by extensive genetic heterogeneity. Recently, three mutations in *SZT2* were reported in two unrelated children with unexplained infantile epileptic encephalopathy with severe ID. Here we report a European American family with three children having non-syndromic mild or moderate ID without seizures. Whole-exome sequencing of three affected siblings revealed a three base pair deletion (c.4202_4204delTTC) located in a 19 mb autozygous region on chromosome 1, leading to an amino acid deletion (p.Phe1401del) in *SZT2*. All three children were homozygous for the deletion and their parents were heterozygous as expected in autosomal recessive inheritance. *SZT2* is highly expressed in neuronal tissues and regulates seizure threshold and neuronal excitation in mice. We conclude that the disruption of *SZT2* with some residual function might lead to mild or moderate ID without seizures.

## Introduction

Recent studies have applied next-generation sequencing technology to identify causative DNA variants in intellectual disability (ID) [1-5]. Here, we report a multiplex European American family with non-syndromic ID and application of whole-exome sequencing to identify the responsible gene, *SZT2*, which was recently reported in two unrelated children with unexplained infantile epileptic encephalopathy with severe ID [6]. 

## Subjects and Methods

This study was approved by the IRB at the University of Miami. Written informed consents were obtained from all participants, or in the case of minors, from the parents. All clinical investigation has been conducted according to the principles expressed in the Declaration of Helsinki. The parents of individuals in this manuscript have given written informed consent (as outlined in PLOS consent form) to publish these case details. DNA sequencing data are stored in a secure internal database, which are available upon request to researchers wishing to use them for research purposes only.

### Studied Family

**Figure 1:**
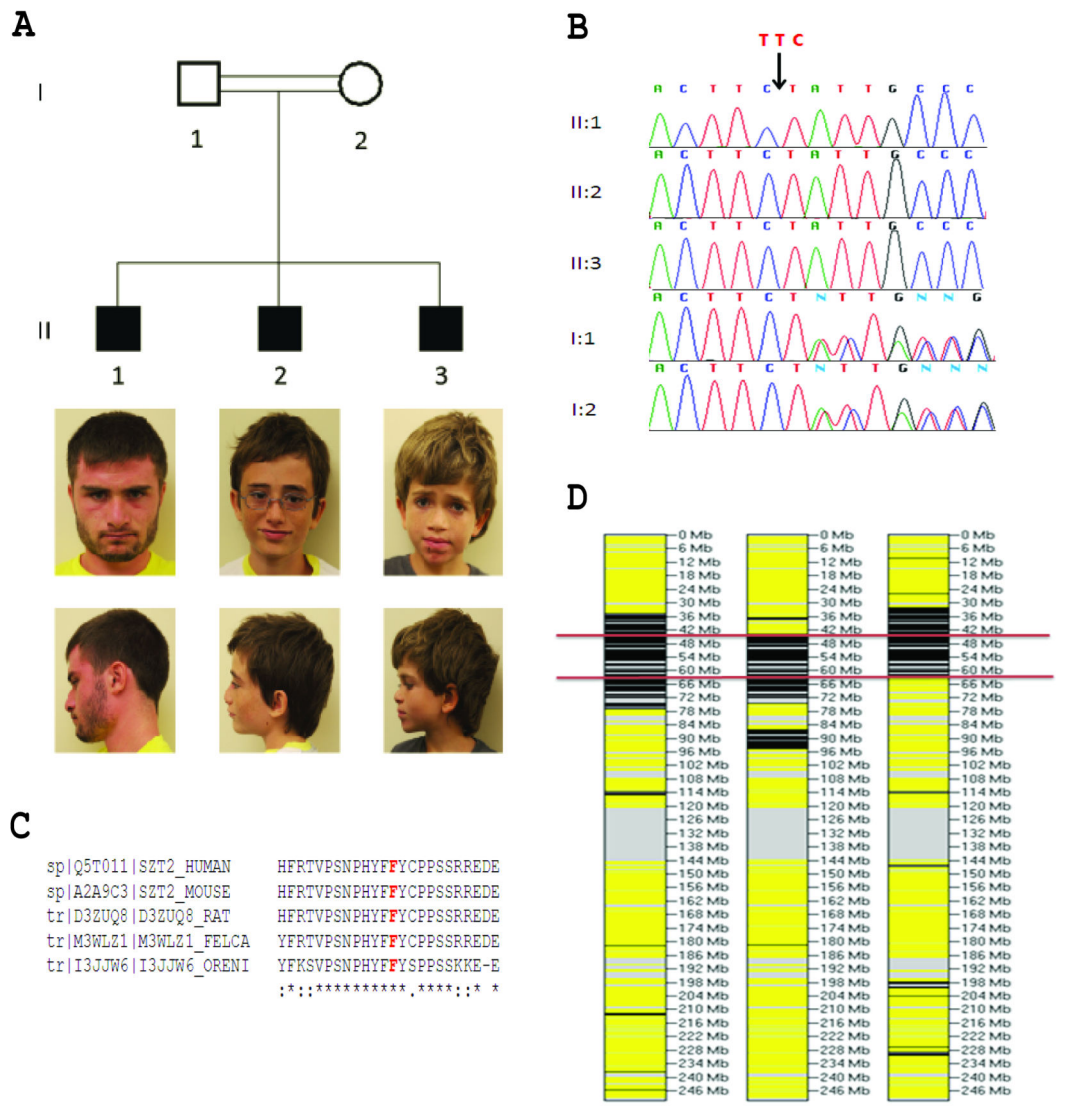
Studied family and identified SZT2 mutation. A) The pedigree of the family and pictures of the affected siblings corresponding to their position in the pedigree. B) Electropherograms showing identified mutation in SZT2 in the family. C) The longest shared autozygous region of 19 mb in three siblings discovered by whole-exome sequencing (on chromosome 1). Black, yellow, and gray denote homozygous, heterozygous, and missing genotypes, respectively. Red lines indicate the borders of the shared autozygous region. D) Conservation of SZT2: Phenylalanine 1401 is conserved among mammals (mouse, rat, house cat) and fish (tilapia).

This study included a family where three brothers with ID were born to healthy consanguineous parents (Figure 1A). Clinical evaluations were performed at the University of Miami and the Comprehensive Neurobehavioral Institute in Plantation, Florida, U.S.A.

### DNA Sequencing

Whole-exome sequencing was conducted using the Agilent SureSelect Human All Exon 50 MB kit and an Illumina HiSeq 2000 instrument. Samples were prepared using standard protocol for the HiSeq2000. 99bp paired-end reads were produced. Sequence reads were developed via the Illumina CASAVA v1.8 pipeline. Alignment of sequence reads to human reference genome (hg19) was completed using BWA, and variants were called using the GATK software package [7-9]. Variants were further characterized using SeattleSeq135.

Analysis of sequencing data was conducted with Genome Management Application (GEMapp), University of Miami Miller School of Medicine (https://genomics.med.miami.edu/). Results were filtered according to autosomal recessive and X-linked modes of inheritance, considering homozygous, compound heterozygous, and hemizygous variants. Evaluation of all missense, nonsense, frameshift, and splice site variants was performed in concurrence with a minor allele frequency <0.01 in both NHLBI (http://evs.gs.washington.edu/EVS/) and dbSNP137 databases. Consideration of a given variant was dependent on absence in >2 families in our internal database, which includes 2,481 whole exomes. Genotypes derived from whole exome data were utilized to identify homozygous runs as previously described [10], where the authors developed two computer programs, *AgileGenotyper* and *AgileVariantMapper* that can identify autozygous regions from an individual's exome sequence data. The two programs differ in that *AgileVariantMapper* uses the genotypes of all positions found ab initio to be polymorphic by the analysis of the exome sequence data, whereas *AgileGenotyper* deduces genotypes at over 0.5 million exonic positions previously found to be polymorphic in the 1000 Genomes Project data set. Autozygosity data developed solely by exome sequencing showed good correlation for the long regions of autozygosity detected by SNP arrays [10]. 

For Sanger sequencing, primers were designed using the software Primer3 v.0.4.0 (http://frodo.wi.mit.edu/). A touchdown protocol was used to amplify target DNA region. The resulting amplicons were cleaned up with Sephhadex (GE Healthcare). ABI PRISM 3130 DNA analyzer (Applied Biosystems) and Big Dye Terminator Cycle Sequencing V3.1 Ready Reaction Kit (Life Technologies) were used to elucidate the DNA sequence. 

## Results

### Clinical Phenotype

The proband (individual II:1 in Figure 1A) is an 18-year-old male who was born to a then 26-year-old mother and 32-year-old father after a normal pregnancy. The parents are first cousins with an ancestral origin in Southern Italy. His perinatal history reveals a C-section delivery with anoxia requiring resuscitation at birth. He had delays in motor, social, and cognitive development during infancy. He has been on medications including atomoxetine, aripiprazole and buspirone along with speech and occupational therapies for several years. Currently, the patient attends a school for the multi-handicapped and receives Exceptional Student Education (ESE) services. His height, weight, and head circumference are 170 cm (between the 25^th^ and 50^th^ centiles), 55 kg (between the 10^th^ and 25^th^ centiles), and 59.6 cm (>97^th^ centile), respectively. The neurological examination is notable for the mental status and behavior. Other systems are unremarkable. The patient understands commands and most simple questions, is often echolalic in his speech, and is mildly agitated. He often needs to be redirected and sometimes restrained, as he is very active and mostly untrainable. He makes adequate eye contact with others, demonstrates affiliative behaviors, and is capable of relating emotionally to those around him through both verbal and non-verbal communication. Two brain MRIs, one at age 10 and one recent, and an EEG are normal. 

Individual II:2 (Figure 1A) is a 10-year-old male born to the same parents as the proband. There was a normal pregnancy and uncomplicated delivery. This child displays a more mild case of developmental delay with inattention. He didn’t begin to speak until 3.5 years of age. He has difficulty learning and retaining new information, is inattentive, and gets easily frustrated. However, he can be controlled by verbal command. Currently he attends school for the ESE students with an estimated IQ at 75. Past medical history is otherwise unremarkable and he is on no medications (parental decision). His height, weight, and head circumference are 135 cm (25^th^ centile), 24 kg (5^th^ centile), and 55.5 cm (97^th^ centile), respectively. The examination is notable for his mental status and behavior. He is mildly to moderately inattentive with normal speech. Both his MRI and EEG are normal.

Individual II:3 (Figure 1A) is a 7-year-old male born to the same parents as the two elder brothers. The pregnancy and delivery were normal. There was no motor delay but speech was delayed, requiring speech therapy. Held back a year, he is now entering first grade and shows signs of inattention and distractibility. The past medical history is negative for concurrent illness and the patient is on no medications (parental decision). Current height, weight, and head circumference measure 116 cm (<3^rd^ centile), 17.5 kg (<5^th^ centile), and 56 cm (>97^th^ centile), respectively. His neurological examination reveals a somewhat shy and reticent child who follows instructions. Though distractible and inattentive, he is easily redirected. Currently, he only speaks in phrases. The remainder of the neurological examination is unremarkable, and his brain MRI is normal. 

Hearing and ophthalmologic examinations were normal in the affected siblings. Chromosomal microarrays, fragile X testing, plasma amino acids, urine organic acids, plasma acylcarnitine profile, and total and free carnitines were also reported to be normal. 

Clinical evaluation of the parents were unremarkable except for an occipitofrontal head circumference of 58.4 cm (>97^th^ centile) and 55.2 cm (between the 75^th^ and 90^th^ centiles) for the father and mother, respectively. 

### An *STZ2* Mutation Co-segregates with ID

Whole-exome sequencing conducted in three affected children II:1, II:2, II:3 generated 80,134,078, 77,777,242, and 74,926,765 sequence reads, respectively. Coverage of targeted regions at 2x and 10x read depth was 98.9% and 88.5% for individual II:1, 98.0% and 86.0% for individual II:2 and 98.0% and 85.1% for individual II:3, respectively. Average quality of single nucleotide variations (SNVs), and insertions and deletions (INDELs) was 1,236 and 1,318 in II:1, 1,282 and 1,367 in II:2, and 1,187 and 1,275 in II:3, respectively. Before filtering the data 85,689 SNVs and 9,248 INDELs were found in II:1, 83,491 SNVs and 8,292 INDELs in II:2 and 83,194 SNVs and 8,323 INDELs in II:3. Filtering for variants shared in all three siblings according to the strategy given in Methods for autosomal recessive and X-linked inheritance yielded only one variant: a homozygous three base pair deletion at position Chr1:43,895,742 bp -43,895,744 bp (hg19), which corresponds to c.4202_4204delTTC in *SZT2* (NM_015284.3) leading to the deletion of a phenylalanine residue (p.Phe1401del) (Figure 1B). The variant showed complete co-segregation with the described phenotype as an autosomal recessive trait and was absent in 2,481 controls from our internal database or in dbSNP137. MutationTaster predicts that this mutation is disease causing (www.mutationtaster.org). The phenylalanine residue, which is deleted, is in a region of the protein that is conserved across species (Figure 1C). The variant was within a 19 mb autozygous region on chromosome 1 shared by three siblings (Figure 1D). Evaluation of the entire genome did not show any other shared autozygous region > 1 mb. There were 138 RefSeq genes with 2,350 exons mapping to the longest autozygous region. 2x coverage for the exonic nucleotides within the autozygous segment was 94% for all three samples; 10x coverage was 93%, 92%, and 92% for individuals II:1, II, and II:3, respectively. 

Entire coding region of chromosome X at 2x and 10x read depth was covered 90% and 87% for individual II:1, 90% and 85% for individual II:2, and 90% and 85% for individual II:3, respectively. Average read depth for chromosome X was 69x, 58x, and 57x for individuals II:1, II:2, and II:3, respectively. Visual evaluation of variants on chromosome X showed that only two regions >500 kb were shared between all three brothers: 12,939,928 bp – 25,014,016 bp and 96,139,459 bp – 142,596,941 bp. X-linked ID without syndromic findings has been previously shown to be caused by mutations in *AP1S2, RPS6KA3, ACSL4, PAK3*, and *ARHGEF6* that mapped to these two shared regions. Whole-exome sequencing covered 100% of coding nucleotides of all these genes with > 2x read depth. 

## Discussion

*SZT2* is a recently identified gene that was shown to cause low seizure threshold and enhance epileptogenesis in mice when mutated [11]. Mutations led to embryonic lethality in some. This gene is expressed in several tissues, but most notably in the brain and also in high levels during embryonic development. With a single ortholog in all land vertebrates and several invertebrates, SZT2 is highly conserved, suggesting the importance of its structure and proper functioning. Additionally, protein databases indicate that SZT2 has no significant sequence similarities to any known protein which prevents precise *in silico* modeling. 

While this manuscript was being prepared, three mutations in *SZT2* were reported in two unrelated children with unexplained infantile epileptic encephalopathy with severe ID [6]. One patient was homozygous for a nonsense mutation and the other was compound heterozygous for another nonsense mutation and a splice-site variant. These mutations were predicted to result in nonsense-mediated mRNA decay and/or premature protein truncation and subsequent complete loss of *SZT2* function. Both patients presented with severe developmental delay, refractory epilepsy, a thick corpus callosum, and persistent cavum septum pellucidum. Therefore, the researchers concluded that mutations in *SZT2* can cause a severe type of autosomal recessive infantile encephalopathy with intractable seizures and distinct neuroradiological anomalies. 

Here we report another *SZT2* mutation that is the homozygous deletion of a single phenylalanine residue. Though the mutation is predicted to cause loss of function, it very likely retains some residual function, which explains a much milder phenotype in these children compared to those with truncating mutations. It is likely that the ID is most pronounced in the proband in this report due to the *SZT2* mutation in combination with the hypoxia he experienced at birth. The other brothers also share the same mutation; however, they did not experience hypoxia at birth suggesting that their relatively milder ID is due entirely to the *SZT2* mutation. Moreover, brain MRIs were normal, epilepsy was not observed in either child, and EEGs were normal in individuals II:1 and II:2. Interestingly, all three children had macrocephaly, suggesting that it is a part of the clinical phenotype caused by the *SZT2* mutation. On the other hand, the unaffected father in our family has macrocephaly and macrocephaly was not present in two children previously reported with *SZT2* mutations. Much about the function of SZT2 still remains unknown, yet our study, as well as the aforementioned studies, reveals that it is vital to proper central nervous system function.
